# Effects of Seven-Year Fertilization Reclamation on Bacterial Community in a Coal Mining Subsidence Area in Shanxi, China

**DOI:** 10.3390/ijerph182312504

**Published:** 2021-11-27

**Authors:** Li Li, Tingliang Li, Huisheng Meng, Yinghe Xie, Jie Zhang, Jianping Hong

**Affiliations:** 1 College of Resources and Environment, Shanxi Agricultural University, Jinzhong 030801, China; lili_306@163.com (L.L.); huishengmeng@126.com (H.M.); xieyinghe@163.com (Y.X.); zhangjie880124@foxmail.com (J.Z.); 2 National Experimental Teaching Demonstration Center for Agricultural Resources and Environment, Shanxi Agricultural University, Jinzhong 030801, China

**Keywords:** coal mining, soil reclamation, bacterial community, bacterial diversity, high-throughput sequencing

## Abstract

The restoration of soil fertility and microbial communities is the key to the soil reclamation and ecological reconstruction in coal mine subsidence areas. However, the response of soil bacterial communities to reclamation is still not well understood. Here, we studied the bacterial communities in fertilizer-reclaimed soil (CK, without fertilizer; CF, chemical fertilizer; M, manure) in the Lu’an reclamation mining region and compared them with those in adjacent subsidence soil (SU) and farmland soil (FA). We found that the compositions of dominant phyla in the reclaimed soil differed greatly from those in the subsidence soil and farmland soil (*p* < 0.05). The related sequences of *Acidobacteria*, *Chloroflexi*, and *Nitrospirae* were mainly from the subsided soil, whereas those of *Alphaproteobacteria*, *Planctomycetes*, and *Deltaproteobacteria* were mainly derived from the farmland soil. Fertilization affected the bacterial community composition in the reclaimed soil, and bacteria richness and diversity increased significantly with the accumulation of soil nutrients after 7 years of reclamation (*p* < 0.05). Moreover, soil properties, especially SOM and pH, were found to play a key role in the restoration of the bacterial community in the reclaimed soil. The results are helpful to the study of soil fertility improvement and ecological restoration in mining areas.

## 1. Introduction

China is one of the largest coal-producing countries in the world [1]. However, the large-scale and high-intensity exploitation of coal resources has caused a series of ecological and environmental problems, such as soil erosion, declines in soil quality, aggravation of land degradation, and imbalance of the soil ecosystem [2,3,4]. In particular, land subsidence caused by underground coal mining can lead to drastic disturbances of soil structure and remarkable variation in soil microbial communities [5,6], which greatly reduce soil fertility, crop productivity, and the stability of the soil ecosystem [4,7,8]. These have serious impacts on the sustainable development of agriculture in mining areas. Land reclamation is an effective method to solve the conflict between coal mining and land resource protection and to alleviate the contradiction between humans and land in the coal mining area.

Restoring soil fertility is the emphasis for land reclamation and ecological restoration in coal mining subsidence areas. Soil microorganisms, one of the most important parts of the soil ecosystem, are essential in soil formation, nutrient cycling (such as carbon, nitrogen, and phosphorus), and ecological balance [9,10,11]. The abundance, diversity, and activity of soil bacteria can be used as effective indicators of soil quality due to their high sensitivity to environmental changes and soil nutrient status [12,13,14]. It has been reported that bacterial community stability in subsidence soil was dramatically disrupted by coal mining activities, resulting in reductions in total bacterial biomass and diversity [15,16,17,18]. To some extent, it is not only necessary to increase soil nutrients but also more important to restore soil microbial activities and communities for the sustainability of reclaimed soil ecosystems [19]. For a long time, research on reclaimed soil has mainly focused on the improvement of soil physicochemical status and vegetation characteristics [20,21]. However, there are few studies on microbial population, diversity, and function in reclaimed soil.

The sustainability of terrestrial agroecosystems depends to a great extent on soil bacterial diversity for sustaining soil biological activity and crop productivity [22,23]. Fertilization can effectively improve soil nutrient conditions and affect soil microbial communities [24,25]. Generally, the application of organic fertilizer is beneficial to soil microbial communities [26,27,28], while the long-term application of chemical fertilizer can decrease soil microbial diversity in farmland [24,29,30]. At present, fertilization is the most effective way to restore cultivated land and improve soil in mining subsidence areas. Different fertilization methods have different effects on soil physical and chemical properties and microbial community. Therefore, an in-depth study on the changes in microbial community composition in reclaimed soil caused by fertilization will help to further understand how soil fertility affects the changes in microbial communities during the restoration of disturbed land to farmland.

The present study was carried out to investigate the response of bacterial communities to land reclamation with different fertilizers. Illumina high-throughput sequencing technology was used to compare the bacterial community structure and diversity in reclaimed soil with those in adjacent unreclaimed soil (from adjacent farmland and subsided land) from a mining area (in the same edaphic-climatic area) in Shanxi Province, China. We hypothesized that fertilization activity in the process of reclamation could improve soil nutrients and increase bacterial community diversity, and the variation of bacterial community structure may be related to changes in physicochemical properties, such as pH, soil organic matter, available nitrogen, phosphorus, and potassium. This study could identify an effective and appropriate method for the rapid restoration of soil fertility and provide a valuable reference for the soil restoration of coal mining subsidence areas with similar climatic and soil conditions.

## 2. Materials and Methods

### 2.1. Experimental Site Description

The field experimental area is located in the Lu’an coal mine (36°28′12′′ N, 113°00′53′′ E), Xiangyuan county in Shanxi, China. This region has a warm and semi-humid continental monsoon climate with a frost-free period of 160 d. The average annual temperature is about 9.5 °C, with monthly mean minimum temperatures occurring in January (−8.1 °C) and monthly mean maximum temperatures in July (23.4 °C). The mean annual precipitation is approximately 532.8 mm and mainly occurs from July to September. The soil type of the research area is calcareous cinnamon soil with silty loam, which is classified as Luvisols according to the World Reference Base (FAO) system [31]. In this region, coal mining has triggered the goaf in underground mines and formed land subsidence since the 1970s. Land consolidation, including topsoil stripping, land leveling, and backfilling, was carried out using large loaders before the reclamation in the autumn of 2008. The leveled land was divided into separate plots for fertilizer reclamation.

### 2.2. Experimental Treatments and Soil Sampling

The field experiment in this reclaimed site included three fertilization amended treatments: without fertilizer (CK), chemical fertilizer (CF), and manure (M) treatment. The chemical fertilizer was applied as urea, calcium superphosphate, and potassium chloride in CF. Decomposed chicken manure (27.8% organic matter, 1.68% N, 1.54% P_2_O_5_, and 0.82% K_2_O) was provided as an organic amendment at a rate of 12,000 kg∙ha^−1^ in the M treatment. An equal amount of 201 kg N∙ha^−1^, 185 kg P_2_O_5_∙ha^−1^, and 98.5 kg K_2_O∙ha^−1^ was applied before corn sowing in the fertilizer treatments. The treatments were arranged in a randomized complete block design with three replicates, and the size of each plot was 100 m^2^. Maize (*Zea mays L.*) was continuously planted in each plot from 2009 to 2015. According to local farming practices, crops are sown on or about May 1 and harvested on October 1. In addition, two unreclaimed treatments (SU and FA) were selected as controls for the reclaimed site. SU is a neighboring unclaimed subsidence site, and its surface vegetation is sparse and naturally growing weed. FA is another adjacent farmland that has been disturbed by coal mining and has been planting maize using local traditional fertilizing practices for many years.

Soil samples were collected after the maize harvest in October 2015. A total of fifteen individual soil samples (5 treatments × 3 replicates) represent three reclaimed treatments (CK, CF, and M) and two controls (SU and FA). All the soil samples were taken using a hand auger (5 cm diameter) at a depth of 0–20 cm after the superficial vegetation was removed, and each sample was a composite of six subsamples randomly collected from the five treatments. After mixing thoroughly, the homogeneous composite soil samples were enclosed in sterile plastic bags and transferred to the laboratory on ice. The samples were sieved through a 2.0 mm mesh and immediately divided into two parts: one part was stored at −80 °C for further molecular analysis, and the other was air-dried for chemical determination.

### 2.3. Selected Soil Properties Analysis

Soil pH was measured with a soil–water mixture (1:1) using a glass combination electrode [32]. Soil organic matter (SOM) was measured according to the method described by Strickland and Sollins [33]. The Mason-jar diffusion method by Bremner [34] was used to determine the soil alkali-hydrolyzable nitrogen (AN). Available phosphorus (AP) was analyzed by resin extraction following a protocol modified from Hedley and Stewart [35]. Available potassium (AK) was extracted with ammonium acetate and determined by flame photometry [36].

### 2.4. DNA Extraction, PCR Amplification, Illumina MiSeq Sequencing, and Sequencing Data Processing

Soil microbial DNA was extracted from approximately 1 g of soil samples using the TIANamp Genomic DNA Kit (TIANGEN Biotech, Beijing, China, Cat. No.: DP304) according to the manufacturer’s instructions. The integrity of the extracted DNA was assessed by agarose gel electrophoresis (1%), and the concentration and purification of the DNA (2 μL) were determined using NanoDrop ND-1000 microspectrophotometry (NanoDrop Technologies, Wilmington, DE, USA). The bacterial primer set of forward primer 341F (5′-CCTACGGGNBGCASCAG-3′) and reverse primer 806R (5′-GACTACNVGGGTATCTAATCC-3′) was used to amplify the 16S rDNA gene sequence in the V3–V4 hypervariable region (465 bp). PCRs were carried out in triplicate, 25 μL reactions with 2.5 μL of Ex Taq buffer (Takara Bio Inc., Kusatsu, Japan, Takara code: RR001B), 1.5 μL of 2.5 mM Mg^2+^, 2 μL of 2.5 mM dNTPs, 0.25 μL Ex Taq DNA Polymerase (Takara Bio Inc., Kusatsu, Japan, Takara Code: RR001B), 16.75 μL of double-distilled water, 10 μM of each primer, and approximately 20 ng of DNA template. The amplification program consisted of an initial denaturation step of 94 for 2 min, followed by 30 cycles of denaturation at 94 for 30 s, annealing at 50 for 30 s, and elongation at 72 for 30 s, with a final extension at 72 for 5 min. Replicate reaction mixtures of the same sample were assembled within a PCR tube. After visualization on agarose gels (1% in TBE buffer) containing ethidium bromide, PCR products were purified using the QIAquick PCR Purification Kit (QIAGEN, Hilden, Germany, Cat. No.: 28106) and quantified with a NanoDrop ND-1000 spectrophotometer (NanoDrop Technologies, Wilmington, DE, USA). Purified amplicons were pooled in equimolar concentrations and paired-end sequenced on the Illumina MiSeq TM System platform according to the manufacturer’s protocols.

Sequence analysis was conducted using quantitative insights into the microbial ecology (QIIME) pipeline (version 1.7.0), as previously described by Fadrosh et al. [37]. After the barcodes and primers were trimmed, and low-quality sequences were removed (<Q20), the remaining high-quality reads were clustered into operational taxonomic units (OTUs) based on their sequence similarity at 97%. Community richness and diversity indices based on the number of OTUs and rarefaction curves were obtained using the Mothur software (version 1.34.0, Pat Schloss, Michigan, USA). Prior to the data analysis, the alpha-diversity indices of the bacterial community, including Good’s coverage, Chao1, ACE, and the Shannon index, were calculated based on an appropriate subsample depth.

### 2.5. Statistical Analysis

Statistical analyses were performed by one-way analysis of variance (ANOVA) using the PASW Statistics program (version 18.0 for windows). The means were segregated using Duncan’s multiple comparison test with a significance level of *p* < 0.05. Pearson’s correlation analysis was conducted to evaluate the correlations between soil physicochemical and microbiological characteristics. To compare bacterial community structures across all soil samples, principal coordinate analysis (PCoA) and cluster analysis were performed based on the unweighted UniFrac distance matrix [38]. Redundancy analysis (RDA) was carried out to examine the relationship between abundant phyla (proteobacterial classes) and soil physicochemical characteristics [39].

## 3. Results

### 3.1. Soil Properties

The result showed that fertilizer reclamation clearly affected the soil nutrient amounts. As shown in Table 1, the subsidence soil showed the lowest nutrient amounts. The amounts of SOM in the SU treatment were 36.56% of that in the FA treatment, and AN, AP, and AK in the SU treatment accounted for 33.97%, 16.37%, and 61.14%, respectively. After the 7-year fertilizer reclamation, the amounts of SOM, AN, AP, and AK were consistently increased in the reclamation-treated soil compared with the subsidence soil. The SOM and AN amounts in the CF and M treatments were significantly higher than those in the CK treatment (*p* < 0.05). Significantly higher SOM and AN amounts (*p* < 0.05) were obtained in the M treatment compared with the CF treatment. The highest pH was observed in the subsidence soil, and fertilizer reclamation decreased the soil pH (Table 1). However, there was no significant difference in soil pH between CF treatment and M treatment.

### 3.2. Soil Bacterial Abundance and Diversity

**Figure 1:**
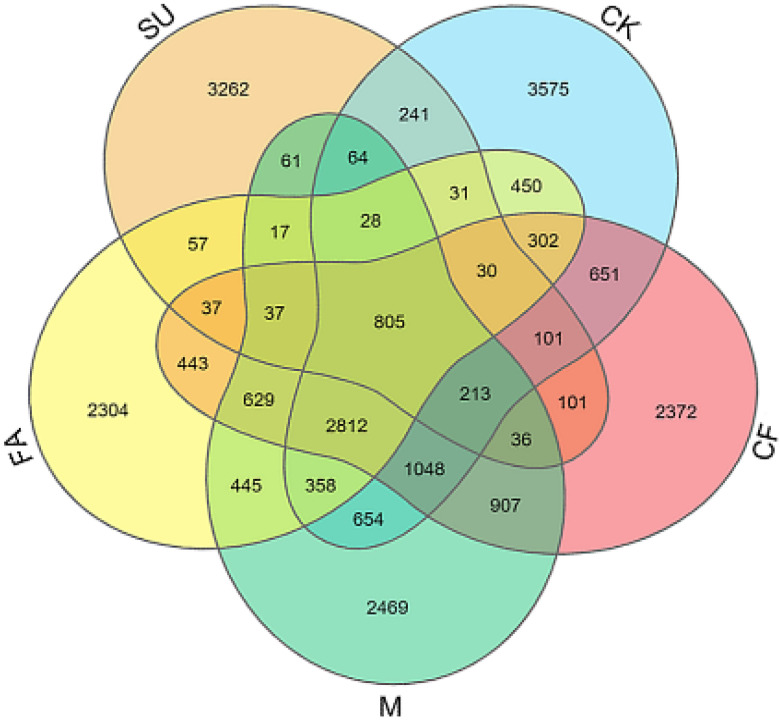
OTU Venn analysis of different treatments. OTU: Operational Taxonomic Units; CK: reclaimed soil sampled in no-fertilizer treatment; CF: reclaimed soil sampled in chemical fertilizer treatment; M: reclaimed soil sampled in manure treatment. SU: subsided soil sampled in an adjacent site; FA: soil sampled in another adjacent farmland.

A total of 138,894 high-quality sequences (average read length of 440 bp) were obtained from all the soil samples. These optimized sequences were clustered into OTUs by Mothur software. As shown in Table 2, the Sobs values in all treatments were in the range of 3672–11,030, and the lowest Sobs was found in the SU treatment. There was no significant difference in Sobs between the reclaimed soil (CK, CF, and M) and farmland soil (FA), but they were significantly higher than those in the subsided soil (SU). Venn analysis (Figure 1) showed that 805 Sobs were found in all five treatments, accounting for 7.2–21.92% of each treatment, respectively. A total of 3478 Sobs were detected in the CK, CF, and M treatments, which were 31.53–35.59% of their total number. In addition, 5131 Sobs were detected in the CF and M treatments, accounting for 46.52% and 46.56% of their total Sobs, respectively. The unique Sobs out of all the treatments accounted for 21.52–88.82%, indicating that there were differences in the composition of the soil bacterial community among the treatments.

The result of bacterial community diversity is presented in Table 2. The SU treatment showed the lowest Chao1, ACE, and Shannon index (alpha-diversity indices), which were significantly different from those in the reclaimed soil. After reclamation, soil bacterial diversity and abundance in the reclaimed soil were significantly higher than those in the subsidence soil (*p* < 0.05). However, Chao1 and ACE showed no significant difference among the CF, M, and FA treatments, which were significantly higher (*p* < 0.05) than those in the CK treatment. The Shannon index of the FA treatment was the highest (11.77) and significantly higher than those of the reclamation treatments (CK, CF, and M). In addition, the Shannon–Weiner curve showed similar trends (FA > CF > M > CK > SU) in terms of high species richness at 97% similarity (Figure S1). Good’s coverage values in all samples ranged from 91% to 97% at a similarity cutoff of 97%, indicating that the current numbers of sequence reads were sufficient to capture the bacterial diversity in these soils.

### 3.3. Soil Bacterial Taxa Community Composition

**Figure 2:**
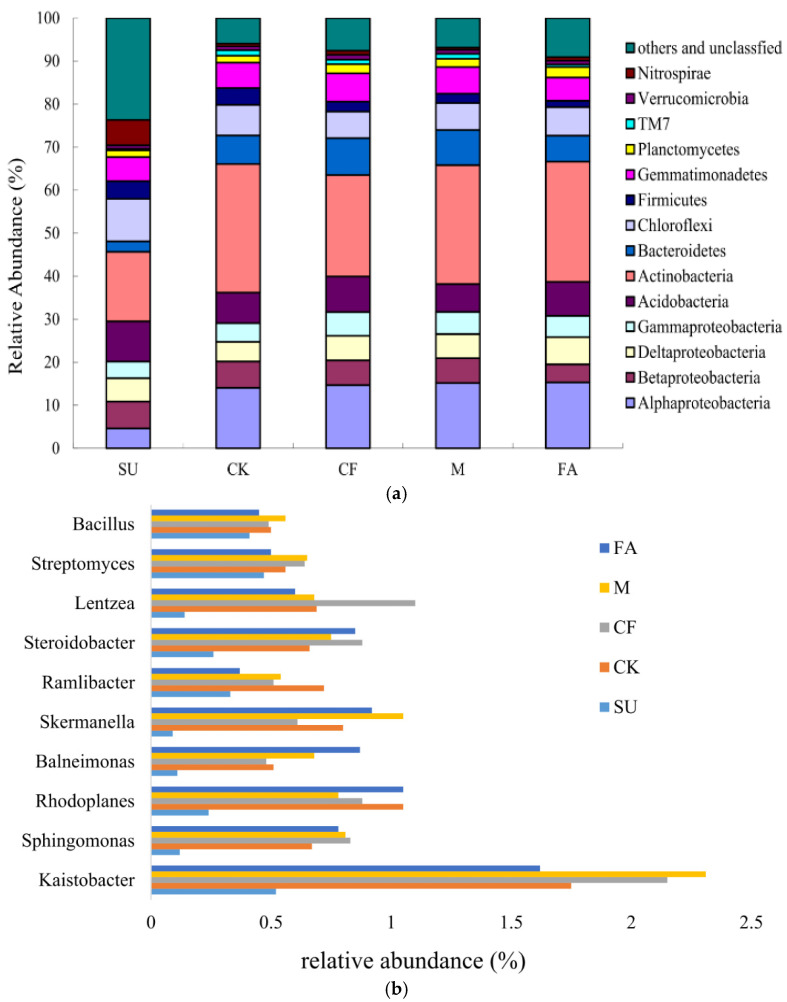
(**a**) Relative abundance of the dominant bacteria phyla (top 10) in all different treatments; (**b**) relative abundance of the dominant bacteria genera (top 10) in all different treatments. Relative abundances (>1%) are based on the proportional frequencies of those DNA sequences that could be classified at the phylum (proteobacterial class) level. Sequences not classified to any known phylum and phylogenetic groups accounting for ≤1% of all classified sequences are summarized in the artificial group “others and unclassified”. CK: reclaimed soil sampled in no-fertilizer treatment; CF: reclaimed soil sampled in chemical fertilizer treatment; M: reclaimed soil sampled in manure treatment. SU: subsided soil sampled in an adjacent site; FA: soil sampled in another adjacent farmland. TM7: the phylum candidatus *Saccharibacteria*.

Based on the Illumina platform analysis, sequences from all soil samples were classified into 54 different phyla, 138 classes, 213 orders, 244 families, 423 genera, and 218 species (Figure S2). Proteobacteria were the most abundant phyla, accounting for 20.15%–31.66% in these five treatments (Figure 2a). Furthermore, classes of *Alphaproteobacteria*, *Betaproteobacteria*, *Deltaproteobacteria*, and *Gammaproteobacteria* were detected in this study, and *Alphaproteobacteria* was the most abundant phylum, accounting for 41.48% of total *Proteobacteria* sequences. Other predominant phyla were *Actinobacteria* (27.80%), *Acidobacteria* (7.96%), *Bacteroidetes* (7.42%), *Chloroflexi* (7.21%), and *Gemmatimonadetes* (6.62%), accounting for 81.30% of the bacterial sequences. Additionally, *Firmicutes* (2.81%), *Planctomycetes* (2.01%), *Cyanobacteria* (1.50%), *Nitrospirae* (1.25%), *TM7* (1.07%), and *Verrucomicrobia* (1.01%) were present in soil samples with lower relative abundances, which occupied 9.65% of bacterial sequences (Figure S2 and Figure 2a).

The distribution of predominant bacterial phyla (classes) between the different treatments is illustrated in Figure 2a. Although similar main phyla (classes) existed in the selected treatments, the relative abundance of taxa in these treatments was different. No significant differences in the abundance of *Gemmatimonadetes* and *Verrucomicrobia* were observed in all treatments (Table S1). The highest relative abundance of *Acidobacteria* (9.37%), *Chloroflexi* (9.85%), and *Nitrospirae* (5.88%), as well as the lowest abundance of *Alphaproteobacteria* (4.16%), *Gammaproteobacteria* (3.85%), and *Actinobacteria* (16.12%), was detected in the SU treatment. *Alphaproteobacteria* (15.32%), *Planctomycetes* (2.44%), and *Deltaproteobacteria* (6.34%) showed the highest relative abundance in the FA treatment, while *Firmicutes* showed the lowest abundance. In the reclaimed soil, *Bacteroidetes* and *Firmicutes* were remarkably distinct (*p* < 0.05) in the CK treatment as compared with those in the CF and M treatments. Moreover, the respective abundances of the top 10 genera were examined to compare the distribution of bacterial genera in the different treatments (Figure 2b). It was found that the most bacterial taxa at the phylogenetic of genera differed greatly among the selected treatments, except for *Streptomyces* and *Bacillus* (Table S1). The lowest (*p* < 0.05) relative abundance of *Sphingomonas* (0.12%), *Rhodoplanes* (0.24%), *Skermanella* (0.09%), *Steroidobacter* (0.24%), and *Lentzea* (0.14%) was obtained in the SU treatment. The abundance of *Kaistobacter* in the M treatment (2.42%) was significantly (*p* < 0.05) higher than that in the FA treatment (1.62%). The highest (*p* < 0.05) abundance of *Lentzea* (1.16%) and *Balneimonas* (0.94%) was observed in the CF and FA treatments, respectively. Furthermore, the abundance of *Balneimonas* and *Lentzea* were significantly distinct (*p* < 0.05) between the CF and M treatments.

**Figure 3:**
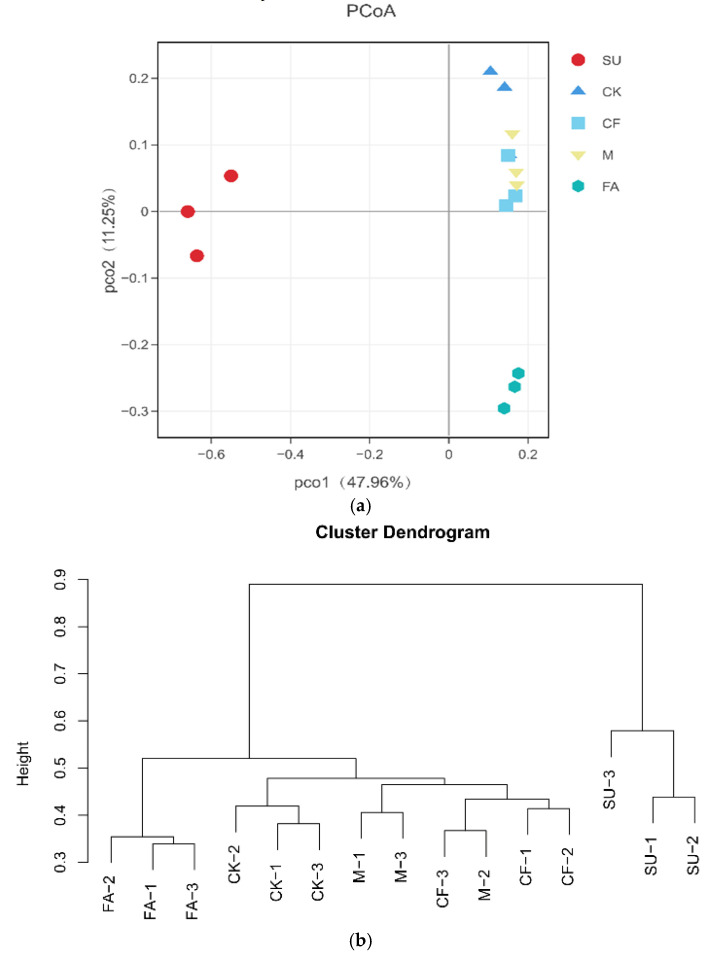
(**a**) Principal coordinate analysis (PCoA) based on unweighted UniFrac distances of soil bacterial communities sampled from different treatments; (**b**) similarity trees based on Bray–Curtis distance indices were calculated by OTUs at a distance of 3% using the hierarchical clustering analysis of bacterial communities for soil samples. CK: reclaimed soil sampled in no-fertilizer treatment; CF: reclaimed soil sampled in chemical fertilizer treatment; M: reclaimed soil sampled in manure treatment. SU: subsided soil sampled in an adjacent site; FA: soil sampled in another adjacent farmland.

PCoA analysis based on the unweighted UniFrac distance metric was conducted to estimate β-diversity, which clearly revealed the variation of bacterial community among the treatments (Figure 3a). The first and second principal components explained 47.96% (PC1) and 11.25% (PC2) of the variance, respectively. As illustrated in the results of PCoA, the soil bacterial community in the SU treatment (with lower soil nutrient content) was separated from that in the CK, CF, M, and FA treatments (with higher soil nutrient content) along the PC1 axis. The soil bacterial community in the farmland soil (with higher soil OM, AN, and AP) was separated from that in the reclamation soil (CK, CF, and M treatments) along the PC2 axis. In addition, the CF and M treatments were clustered together and located in the first quadrant. It meant that the soil from the CF and M treatments had some of the same Sobs but at different levels. Moreover, Bray–Curtis analysis of dissimilarity (ANOSIM, R = 0.778, *p* = 0.001) showed that the differences between groups were larger than within groups, thus there was dissimilarity between the groups.

A hierarchical cluster analysis based on the beta distance matrix was conducted to compare the similarity of the soil bacterial community in the different treatments. The results showed that soil samples (three replicates) from the subsidence site were grouped together and differed from other samples (Figure 3b). The other samples were clustered into two main groups: one group consisting of reclaimed soil samples from the CK, CF, and M treatments and the other consisting of samples from the FA treatment. In addition, the cluster tree revealed that the bacterial communities in the soil of the CF treatment were similar to that of the M treatment, which was different from that of the CK treatment. Overall, the results of cluster analysis were in line with PCoA.

### 3.4. Relationship between Soil Properties and Bacterial Community Composition

The results of Pearson’s correlation analysis showed that soil properties were highly related to bacterial abundance and diversity. As shown in Table 3, soil pH showed significant negative correlations with Chao1 (*r* = −0.826, *p* < 0.01), ACE (*r* = −0.824, *p* < 0.01), and Shannon index (*r* = −0.745, *p* < 0.01). In contrast, SOM, AN, and AP showed significant positive correlations with Chao1 (*r* = 0.545–0.606, *p* < 0.05), ACE (*r* = 0.564–0.659, *p* < 0.05, *p* < 0.01), and Shannon index (*r* = 0.559–0.623, *p* < 0.05). AK was positively correlated with Chao1 (*r* = 0.644, *p* < 0.01) and ACE (*r* = 0.656, *p* < 0.01).

**Figure 4:**
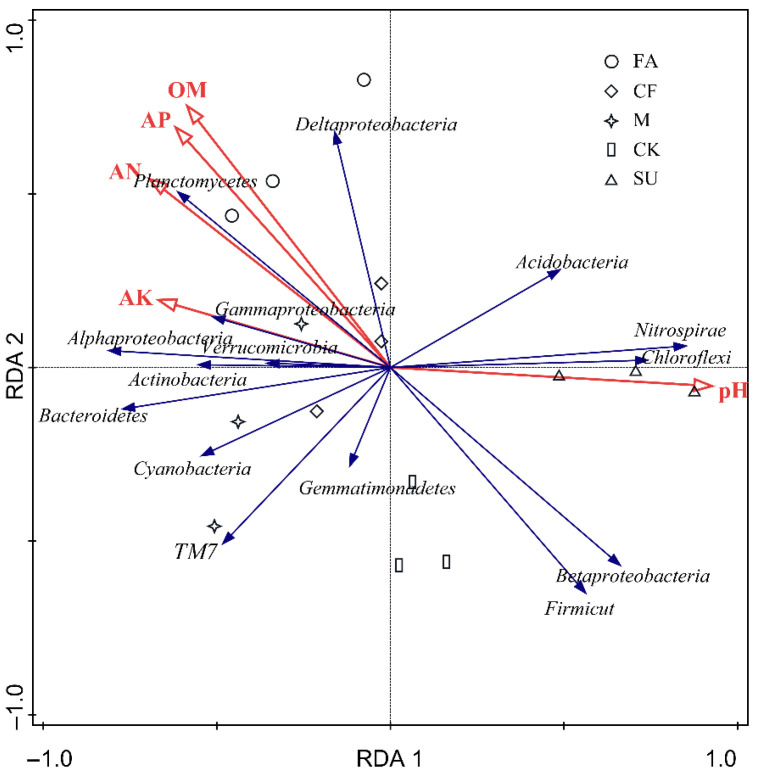
Redundancy analysis (RDA) of abundant phyla (*proteobacterial* classes) and selected soil edaphic properties such as pH, SOM, AN, AP, and AK for individual samples from three sites. SOM: organic matter; AN: alkali-hydrolyzable nitrogen; AP: available phosphorus; AK: available potassium; CK: reclaimed soil sampled in no-fertilizer treatment; CF: reclaimed soil sampled in chemical fertilizer treatment; M: reclaimed soil sampled in manure treatment. SU: subsided soil sampled in an adjacent site; FA: soil sampled in another adjacent farmland. TM7: the phylum candidatus *Saccharibacteria*. The red arrow: soil property factors; The blue arrow: the main group of bacteria phyla.

Results of the RDA analysis are shown in Figure 4. Selected soil property factors could explain 78.96% of the variation. The first and second axes of RDA explained 64.4% and 9.6% of total variation in our data, respectively. The bacterial community of the FA treatment was related to higher SOM, AN, and AP contents and lower pH, as shown by the vectors, while that of the SU treatment was associated with higher pH and lower soil nutrients (SOM, AN, and AP). As shown in Figure 4, the main group of phyla in the CK, M, and CF treatments was closer to that in the FA treatment along the first axis with decreasing pH and increasing soil nutrient contents. Furthermore, abundant phyla of the M and CF treatments were more alike their closer distance than the other treatments. The contributions of the selected physicochemical factors followed this trend: pH > SOM > AP > AN > AK, and their contributions were 39.34%, 27.59%, 18.27%, 13.80% and 11.27%. It is indicated that the structure of the bacterial community was closely correlated with soil properties and mainly shaped by soil pH and SOM. Pearson’s correlation was calculated between the most abundant bacterial phyla (*Proteobacteria* classes) and soil environmental factors (Table S2). We found that the relative abundance of dominant phyla (*Proteobacteria* classes), such as *Alphaproteobacteria*, *Gammaproteobacteria*, *Bacteroidetes*, and *Planctomycetes*, showed significantly negative correlations with soil pH and positive correlations with AK (*p* < 0.05; *p* < 0.01). *Chloroflexi*, *Firmicutes*, and *Nitrospirae* showed significantly positive correlations with pH and negative correlations with SOM, AN, and AK (*p* < 0.05; *p* < 0.01). *Alphaproteobacteria* and *Planctomycetes* were positively correlated with SOM and AN (*p* < 0.05; *p* < 0.01), and *Actinobacteria* was positively correlated with AN (*p* < 0.05). Additionally, *Acidobacteria*, *Gemmatimonadetes*, *Verrucomicrobia*, and *TM7* had no significant correlation with the selected soil factors.

## 4. Discussion

### 4.1. Effects of Reclamation on Soil Properties and Bacterial Community

Coal mining disturbance and reclamation can change original soil physical and chemical characteristics, consequently affecting soil microbial community structure and diversity [16,40,41]. In general, coal mining activity and preliminary engineering reclamation, including stripping, reconstruction, and tillage, lead to the disturbance of vegetation and original surface soil structure, resulting in the degradation of soil bacterial diversity. In our study, the lower soil nutrients and bacterial community diversity shown in the subsided soil were mainly attributable to the deficient management and high erosion rates in the post-mined soil. After 7-year consecutive fertilizer reclamation, *Proteobacteria*, *Actinobacteria*, *Acidobacteria*, *Bacteroidetes*, *Chloroflexi*, and *Gemmatimonadetes* were the main bacterial phyla (Figure S2). In particular, *Proteobacteria* and *Actinobacteria*, as the copiotrophic groups living in nutrient-rich conditions, were the most dominant phyla in the reclaimed soil (Figure 2a), which is in agreement with previous studies [42,43,44]. This consistency of dominant phyla in different mining areas indicates that these bacteria play an important role in soil improvement in mining areas and have a wide range of adaptability to the soil environment of the mining area.

In this study, we found *Alphaproteobacteria* was the largest subgroup of *Proteobacteria* in the reclaimed soil. Moreover, the populations of *Rhizobiales*, *Rhodospirillales*, and *Sphingomonadales* affiliated with the alpha-subclass were also observed in significant proportions in the reclaimed soil (Table S1). The bacterial community of those functional species played an important role in C, N cycles and in maintaining the integrity of the coal mine ecosystem [45]. This is consistent with our findings of higher SOM and AN in the reclaimed soil. In contrast, we found *Chloroflexi* (9.85%) was the advantageous population in the subsidence soil (Table S1) and showed lower abundance in the reclaimed soil with improvement in soil nutrients. This is mainly because this bacterium is a kind of autotrophic bacterium that does not depend on the nutrient supply in the environment and has a survival advantage in barren soil [32]. We also found a higher abundance of *Acidobacteria* (9.37%) in the subsidence soil (Table S1). As a type of slow-growing oligotroph [46] with a rich diversity of metabolism and function, they prefer oligotrophic living environments with poor available carbon sources [47]. Our results demonstrated that the accumulation of soil available nutrients promoted copiotrophic bacteria but negatively affected the oligotrophic groups [8].

Reclamation significantly increased the nutrient contents (SOM, AN, AP, and AK) of reclaimed soil, improved the abundance and diversity of bacteria, and promoted the restoration of the soil microbial community in the mining area [2,16,18,48]. In the present study, bacterial communities were restored after 7 years of reclamation with the increase in soil fertility, but it still differed from the adjacent farmland soil. In the reclaimed soil, the bacterial community composition of the organic fertilizer and chemical fertilizer treatments showed higher similarity (PCoA and cluster analysis). We found that *Actinomycetes* in manure treatment were slightly more abundant than those in the chemical fertilizer treatment (Table S1). Furthermore, *Lentzea* (affiliated to *Actinomycetales*) in the manure treatment were also significantly higher than those in the chemical fertilizer treatment (Table S1). This may be because manure contains more carbon and nitrogen sources for the growth of *Actinomycetes* than chemical fertilizers. In addition, the organic matter in manure can improve the aggregation and water-holding capacity of the soil, which is beneficial to the growth of *Actinomycetes*. We also observed that soil treated with manure had a higher abundance of *Balneimonas* (affiliated with *Rhizobiales*) than soil treated with chemical fertilizer (Table S1), probably because the dramatic increase in organic matter in manure could promote the intensive reproduction of this microbial taxon [49].

The results of PCoA and cluster analysis confirmed the distinct difference in bacterial community composition existed between the reclaimed and subsided soil (Figure 3a,b). It should be noted that the CK treatment (planting maize without fertilization for 7 years) significantly increased SOM and AN compared with the SU treatment, which resulted in elevated bacterial richness and diversity indices. This is mainly because the input of previous maize residues and the exudates released by maize roots through consolidation directly provided the energy source for microorganisms [50]. Additionally, aboveground maize can also alter the soil bacterial community by affecting the quality and quantity of microbial metabolic substrates [51]. These results indicated that surface vegetation restoration was also an important factor affecting bacterial communities in the reclaimed soil. However, the influences of various plants on soil microorganisms and soil physicochemical properties are different [52,53]. Different results could be obtained if other plants were selected in the soil reclamation of the mining area in this study.

Fertilization can improve soil quality and accelerate soil maturation, which further affects the diversity and richness of bacteria [25]. The application of organic fertilizer can improve soil structure and function and promote microbial richness and abundance [26,27,28,45], which is consistent with our results. In our study, nutrient contents and bacterial richness (Chao1 and ACE) in soil treated with manure were higher than those treated with chemical fertilizer (Table 1 and Table 2), which may be related to the input of organic matter. In addition, it is probably because the original bacteria from manure contributed to the increase in bacterial species. Conversely, the CF treatment had remarkably higher bacterial diversity (Shannon index) than the M treatment (Table 2), mainly due to the mineral nutrient supplied by chemical fertilizer being sufficient to induce the corresponding bacterial population in the root system and increase the soil bacterial diversity. In addition, a dramatic increase in organic matter contained in mature crops could promote the intensive reproduction of some microbial groups (*Proteobacteria*, *Actinobacteria*, and *Bacteroidetes*), resulting in the decrease in soil bacterial diversity [49]. It has been reported that the long-term application of chemical fertilizer can destroy soil structure, cause soil acidification, reduce soil enzyme activity, and decrease microbial biomass and diversity [24,29,30]. In this study, fertilizers also significantly increased the abundance and diversity of soil bacteria, which may be related to the lower nutrient levels in the soil before fertilization treatment. It should be pointed out that, under equal nutrient conditions, much more manure will be applied than chemical fertilizer because of its fewer available nutrients. Therefore, the detrimental effects of heavy metal accumulation and antibiotic residues on soil bacteria caused by the extensive application of manure [54,55,56] cannot be ignored.

### 4.2. Effects of Soil Properties on Bacterial Community Composition

Soil microorganisms are closely related to soil properties [13,16,25,57]. Many studies have reported that improved soil fertility resulted in higher bacterial abundance and diversity [45,58,59]. In this experiment, we found that Chao1, ACE, and Shannon index were positively correlated with SOM, AN, AP, and AK (*p* < 0.01 or *p* < 0.05). It is indicated that the accumulation of soil organic matter and nutrients could be a good explanation for the higher bacterial richness and diversity indices in the reclaimed soil (Table 3). We also found that most of the abundant phyla or classes were significantly correlated with one or more selected soil properties, emphasizing the critical role of soil organic matter and nutrients in shaping the abundance and diversity of the soil bacterial community.

Previous studies have indicated that pH, SOM, and AN are the main factors affecting the composition of bacterial communities in soil [60,61,62,63], which is consistent with our study. In this study, pH was the major factor affecting the bacterial community structure in the reclaimed soil. After 7 years of reclamation, soil pH was significantly reduced. Moreover, the application of chemical fertilizer and manure decreased the soil pH, but there was no significant difference with no fertilizer (CK). This may be due to the short reclamation time (≤7 years) resulting in the insufficient effect of fertilization on soil pH (Table 1). Furthermore, it may relate to our experimental calcareous soil [8]. In this experiment, bacterial diversity increased with the decrease in soil pH (Table 2). It was found that there was a positive correlation between soil pH and *Nitrospirae* because *Nitrospirae* are sensitive to soil acidity and usually have a high abundance in alkaline soil [8,64]. Moreover, the abundance of *Actinobacteria* and *Bacteroidetes* had a negative correlation with the soil pH (Table S2), indicating that the bacteria were most abundant in agricultural soil near neutral pH [63,64]. It was also found that pH had a closer relationship with Chao1, ACE, and Shannon index (Table 3) than soil nutrients (SOM, AN, and AP) did. It is indicated that the soil bacterial diversity and community structure are shaped more by changes in soil pH than by direct nutrient addition (Zhang et al. 2017). In addition, soil pH could affect the structure of soil bacterial communities by altering many other environmental factors [42,65].

SOM is another major factor that was found to influence the soil bacterial community. It was observed that there were significant positive correlations with SOM, Chao1, ACE, and Shannon index (*p* < 0.05, Table 3). These results suggested that the restoration of bacterial diversity occurred gradually with the accumulation of soil organic carbon. Soil organic matter is considered to be one of the most common indicators of soil quality. The increase in soil organic matter can promote soil aggregation and improve soil physical properties (e.g., soil structure, bulk density, and water storage) and nutrients, which contributed to the growth and restoration of bacteria in the reclaimed soil. In addition, as a reservoir of carbon and nitrogen sources, the successive decomposition of SOM can produce diverse substrates for microbiota, thus contributing to the improvement of bacterial community diversity [45]. It was also pointed out that SOM had a significant positive correlation with the abundance of *Proteobacteria*, *Actinobacteria*, and *Bacteroidetes* (*p* < 0.05, RDA). These copiotrophic bacteria have a higher abundance in reclaimed soil for their fast growth rates in nutrient-rich conditions [46].

The aim of reclamation in the mining land is to re-establish a productive, healthy, and sustainable ecosystem suitable for post-mining land use. The restoration of soil fertility by increasing soil nutrients and enriching microbial populations is an effective method for ecological restoration in mining areas [66]. However, the reestablishment of the soil microbial community in mining subsidence areas not only depends on the reclamation practices but also the reclamation time. In general, soil nutrients are gradually accumulated with the increase in reclamation time, and soil fertility can approach a relatively stable or predisturbance level after almost 20 years of reclamation [2,48,59]. Studies on mining reclamation suggest that the most important recovery phase of microbial community occurs between 5 and 20 years after reclamation, and the difference is mainly associated with several critical factors, including reclamation practice, soil properties, climatic conditions, and vegetation [16,18,51]. In our study, soil nutrient and bacterial community diversity were significantly improved after 7 years of reclamation but did not reach the level of adjacent farmland soil. Continuous monitoring of soil nutrients and microbial communities in selected sites will be still needed in the future.

## 5. Conclusions

In summary, coal mining subsidence greatly reduced soil fertility and changed the bacterial community. Reclamation could promote the soil bacterial diversity and community composition in coal mining areas by improving soil physical and chemical characteristics. According to our hypothesis, the soil bacterial richness and diversity were elevated with the improvement of soil nutrients after 7 years of reclamation, but it still did not reach the level of the adjacent farmland soil. Moreover, fertilization (organic fertilizer and chemical fertilizer) could significantly increase the soil bacterial abundance, while inorganic fertilizer had a more obvious impact on bacterial diversity. In addition, soil physicochemical factors affected by the soil fertilizer remediation, especially SOM and pH, are critical in shaping the main bacterial populations.

## Figures and Tables

**Table 1:** Soil chemical characteristics in different treatments.

Treatments	pH	SOM (g∙kg^−1^)	AN (mg∙kg^−1^)	AP (mg∙kg^−1^)	AK (mg∙kg^−1^)
SU	8.06 ± 0.06 ^a^	9.74 ± 0.28 ^e^	16.51 ± 0.58 ^e^	3.18 ± 0.12 ^c^	123.30 ± 4.61 ^b^
CK	7.91 ± 0.03 ^b^	11.37 ± 0.59 ^d^	24.68 ± 1.01 ^d^	4.65 ± 0.31 ^c^	135.30 ± 2.31 ^b^
CF	7.84 ± 0.02 ^bc^	14.06 ± 0.46 ^c^	27.13 ± 1.46 ^c^	17.75 ± 1.54 ^b^	233.57 ± 15.32 ^a^
M	7.76 ± 0.02 ^c^	18.15 ± 0.51 ^b^	35.60 ± 0.68 ^b^	19.46 ± 1.47 ^b^	236.36 ± 19.58 ^a^
FA	7.85 ± 0.03 ^bc^	26.64 ± 0.40 ^a^	48.59 ± 2.33 ^a^	35.60 ± 0.68 ^a^	201.7 ± 15.78 ^a^

Values followed by different lowercase letters (a–e) are significantly different (*p* < 0.05) according to Duncan’s multiple comparison test; SOM: organic matter; AN: alkali-hydrolyzable nitrogen; AP: available phosphorus; AK: available potassium; CK: reclaimed soil sampled in no-fertilizer treatment; CF: reclaimed soil sampled in chemical fertilizer treatment; M: reclaimed soil sampled in manure treatment. SU: subsided soil sampled in an adjacent site; FA: soil sampled in another adjacent farmland.

**Table 2:** Estimated number of observed Sobs, coverage, richness, and diversity in different treatments.

Treatments	Sobs	Coverage	Richness and Diversity Indices		
			Chao1	ACE	Shannon
SU	3672 ± 195 ^b^	0.97 ± 0.01 ^a^	4455 ± 323 ^c^	4358 ± 319 ^c^	10.02 ± 0.24 ^d^
CK	10803 ± 106 ^a^	0.96 ± 0.02 ^a^	13864 ± 174 ^b^	14078 ± 242 ^b^	11.37 ± 0.05 ^c^
CF	11021 ± 731 ^a^	0.94 ± 0.01 ^ab^	15190 ± 463 ^a^	15621 ± 413 ^a^	11.60 ± 0.05 ^b^
M	11030 ± 337 ^a^	0.95 ± 0.00 ^a^	15330 ± 216 ^a^	15845 ± 170 ^a^	11.39 ± 0.12 ^c^
FA	9772 ± 288 ^a^	0.91 ± 0.02 ^b^	14430 ± 313 ^a^	14988 ± 302 ^a^	11.77 ± 0.04 ^a^

Values followed by different lowercase letters (a–d) are significantly different (*p* < 0.05) according to Duncan’s multiple comparison test; Sobs: the species of OTU that can be detected; Coverage: Good’s nonparametric coverage estimator; ACE: abundance-based coverage estimator; Shannon: nonparametric Shannon diversity index. CK: reclaimed soil sampled in no-fertilizer treatment; CF: reclaimed soil sampled in chemical fertilizer treatment; M: reclaimed soil sampled in manure treatment. SU: subsided soil sampled in an adjacent site; FA: soil sampled in another adjacent farmland.

**Table 3:** Pearson’s correlation coefficients between soil physicochemical characteristics and Sobs, and diversity indices.

Pearson	Sobs	Chao1	ACE	Shannon
pH	−0.768 **	−0.826 **	−0.824 **	−0.745 **
SOM	0.399	0.545 *	0.564 *	0.616 *
AN	0.463	0.572 *	0.647 **	0.559 *
AP	0.377	0.606 *	0.659 **	0. 623 *
AK	0.508	0.644 **	0.656 **	0.409

** Correlation is significant at the 0.01 level; * Correlation is significant at the 0.05 level. pH: potential of hydrogen; SOM: organic matter; AN: alkali-hydrolyzable nitrogen; AP: available phosphorus; AK: available potassium.

## Supplementary Materials

The following are available online at https://www.mdpi.com/article/10.3390/ijerph182312504/s1, Figure S1: Shannon wiener curves of OUT cluster at 97% sequence identity across different soil samples, Figure S2: The composition of the bacterial community taxa in all soil samples. Table S1: Relative abundance of dominant bacterial community taxa in different treatment, Table S2: Pearson’s correlation coefficients between soil properties and dominant bacterial community taxa.
