# Supplementary material for: Effects of Seven-Year Fertilization Reclamation on Bacterial Community in a Coal Mining Subsidence Area in Shanxi, China
Source: Int J Environ Res Public Health. 2021 Nov 27;18(23):12504. doi: 10.3390/ijerph182312504 (PMC8656652; doi:10.3390/ijerph182312504)
Supplement: Supplementary file 1 [file ijerph-18-12504-s001.zip › ijerph-1450957-supplementary.pdf]

Table S1. Relative abundance of dominant bacterial community taxa in different treatment.

Phylum	Class	Order	Family	Genus	Treatments				
					SU	CK	CF	M	FA
Proteobacteria	—	—	—	—	20.15b	29.08a	31.66a	31.70a	30.76a
	Alphaproteobacteria	—	—	—	4.61b	14.04a	14.68a	15.17a	15.32a
		Sphingomonadales	*Sphingomonadaceae*	*Kaistobacter*	0.56c	1.83b	2.28a	2.42a	1.62b
			Sphingomonadaceae	*Sphingomonas*	0.12b	0.67a	0.83a	0.81a	0.78a
		Rhizobiales	*Hyphomicrobiaceae*	*Rhodoplanes*	0.24b	1.08a	0.88a	0.78a	1.05a
			Bradyrhizobiaceae	*Balneimonas*	0.12c	0.54b	0.48b	0.71a	0.94a
		Rhodospirillales	*Rhodospirillaceae*	*Skermanella*	0.09b	0.80a	0.61a	1.05a	0.92a
	Betaproteobacteria	—	—	—	6.25a	6.13a	5.75a	5.76a	4.19b
		Burkholderiales	*Comamonadaceae*	*Ramlibacter*	0.34b	0.74a	0.51ab	0.54ab	0.37b
	Deltaproteobacteria	—	—	—	5.44b	4.56ab	5.72ab	5.63ab	6.34a
	Gammaproteobacteria	—	—	—	3.85b	4.35b	5.51a	5.14a	4.91ab
		Xanthomonadales	*Sinobacteraceae*	*Steroidobacter*	0.24b	0.66a	0.88a	0.75a	0.85a
Actinobacteria	—	—	—	—	16.12b	29.89a	23.56ab	27.67ab	27.95ab
	Actinobacteria	*Actinomycetales*	*Actinosynnemataceae*	*Lentzea*	0.14c	0.72b	1.16a	0.70b	0.65b
			Streptomycetaceae	*Streptomyces*	0.50a	0.58a	0.66a	0.67a	0.50a
Acidobacteria	—	—	—	—	9.37a	7.1ab	8.28ab	6.46b	7.91ab
Chloroflexi	—	—	—	—	9.85a	7.12b	6.18b	6.27b	6.63b
Bacteroidetes	—	—	—	—	2.47c	6.62b	8.59a	8.14a	6.03b
Gemmatimonadetes	—	—	—	—	5.61a	5.90a	6.59a	6.22a	5.42a
Firmicutes	—	—	—	—	4.12a	3.92a	2.29b	2.15b	1.47b
	Bacilli	*Bacillales*	*Bacillaceae*	*Bacillus*	0.41a	0.50a	0.49a	0.56a	0.45a
Nitrospirae	*Nitrospira*	—	—	—	5.88a	0.67b	0.94b	0.52b	0.82b
Planctomycetes	—	—	—	—	1.55b	1.61b	2.12ab	1.92ab	2.44a
Verrucomicrobia	—	—	—	—	0.80a	0.88a	1.15a	0.94a	0.90a
TM7	—	—	—	—	0.38c	1.28a	1.04a	1.15a	0.58b

Values followed by different lowercase letters are significantly different ($P<0.05$) according to Duncan's multiple comparison test.

Table S2. Pearson's correlation coefficients between soil properties and dominant bacterial community taxa.

Phylum	Class	Treatments				
		pH	SOM	AN	AP	AK
Proteobacteria	—	-0.798**	0.505*	0.639*	0.527*	0.599*
	Alphaproteobacteria	-0.772**	0.571*	0.708**	0.449	0.522*
	Betaproteobacteria	0.362	-0.760**	-0.665**	0.093	-0.235
	Deltaproteobacteria	-0.240	0.535*	0.434	0.098	0.311
	Gammaproteobacteria	-0.606*	0.257	0.303	0.403	0.555*
Actinobacteria	—	-0.481*	0.436*	0.557*	0.163	0.339
Acidobacteria	—	0.480	-0.161	-0.324	-0.300	-0.364
Chloroflexi	—	0.716**	-0.573*	-0.561*	-0.486	-0.520*
Bacteroidetes	—	-0.679**	0.234	0.410	0.748**	0.676**
Gemmatimonadetes	—	0.038	-0.342	-0.264	0.501	0.157
Firmicutes	—	0.606*	-0.781**	-0.782**	-0.410	-0.602*
Nitrospirae	*Nitrospira*	0.773**	-0.562*	-0.615*	-0.451	-0.529*
Planctomycetes	—	-0.630*	0.580*	0.585*	0.296	0.391
Verrucomicrobia	—	-0.369	-0.024	0.012	0.369	0.267
TM7	—	-0.320	-0.275	-0.087	0.451	0.260

** Correlation is significant at the 0.01 level; * Correlation is significant at the 0.05 level.
