# Supplementary figures and images for: Effects of Seven-Year Fertilization Reclamation on Bacterial Community in a Coal Mining Subsidence Area in Shanxi, China
Source: Int J Environ Res Public Health. 2021 Nov 27;18(23):12504. doi: 10.3390/ijerph182312504 (PMC8656652; doi:10.3390/ijerph182312504)

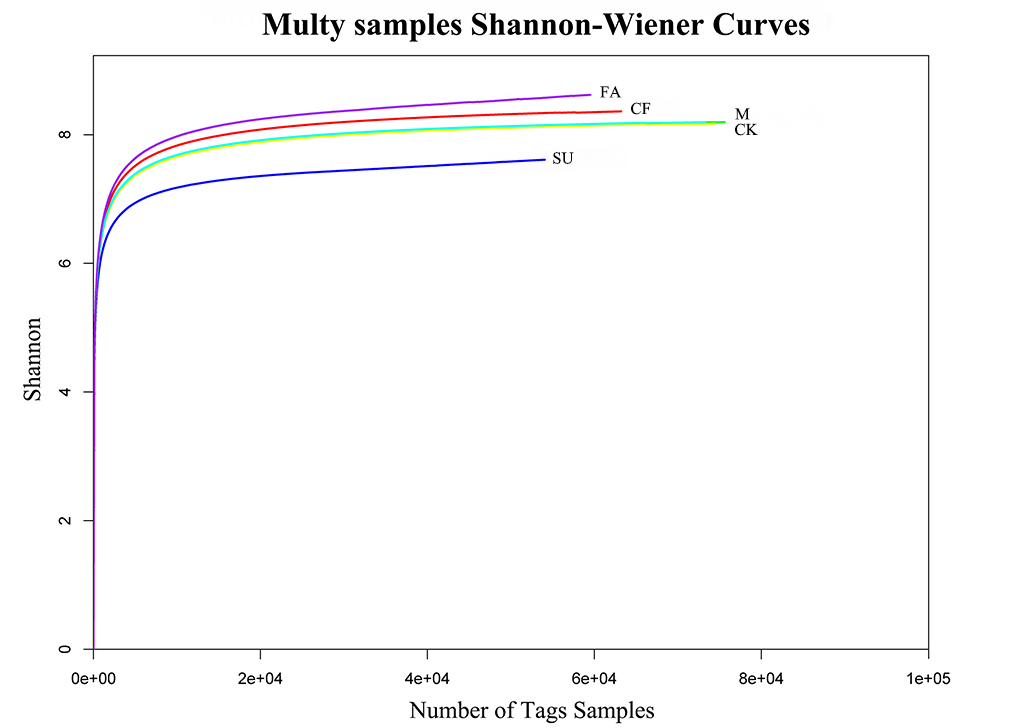

Supplement: Supplementary file 1 [file ijerph-18-12504-s001.zip › fig.S1.tif]

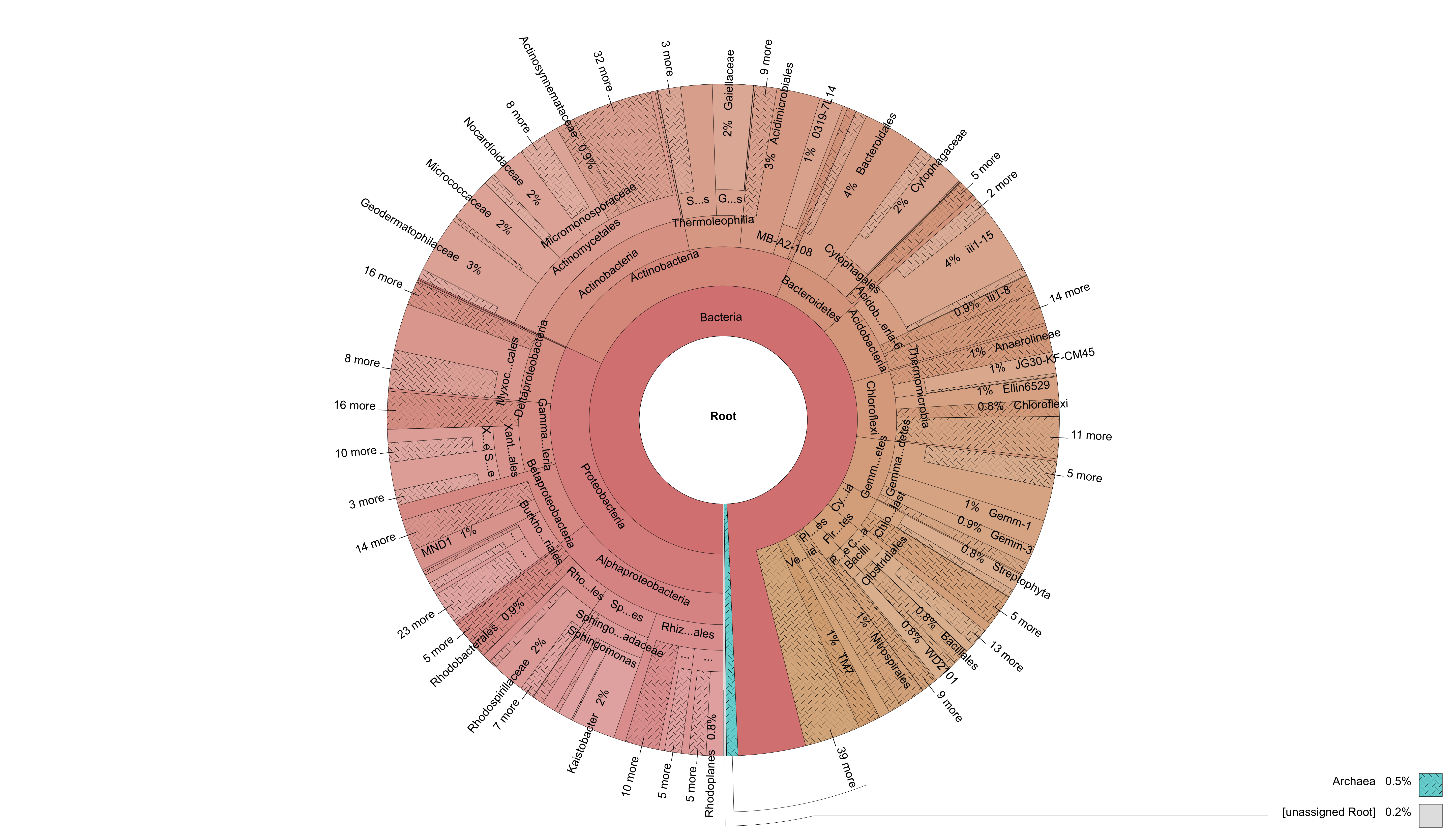

Supplement: Supplementary file 1 [file ijerph-18-12504-s001.zip › Fig.S2.tif]
